# Evidence that genetic drift not adaptation drives *fast‐Z* and *large‐Z* effects in *Ficedula* flycatchers

**DOI:** 10.1111/mec.17262

**Published:** 2024-01-09

**Authors:** Madeline A. Chase, Maurine Vilcot, Carina F. Mugal

**Affiliations:** ^1^ Department of Ecology and Genetics Uppsala University Uppsala Sweden; ^2^ Swiss Ornithological Institute Sempach Switzerland; ^3^ CEFE, University of Montpellier, CNRS, EPHE, IRD Montpellier France; ^4^ Laboratory of Biometry and Evolutionary Biology University of Lyon 1, CNRS UMR 5558 Villeurbanne France

**Keywords:** gene flow, hybrid incompatibilities, reproductive isolation, sex chromosomes, speciation

## Abstract

The sex chromosomes have been hypothesized to play a key role in driving adaptation and speciation across many taxa. The reason for this is thought to be the hemizygosity of the heteromorphic part of sex chromosomes in the heterogametic sex, which exposes recessive mutations to natural and sexual selection. The exposure of recessive beneficial mutations increases their rate of fixation on the sex chromosomes, which results in a faster rate of evolution. In addition, genetic incompatibilities between sex‐linked loci are exposed faster in the genomic background of hybrids of divergent lineages, which makes sex chromosomes contribute disproportionately to reproductive isolation. However, in birds, which show a Z/W sex determination system, the role of adaptation versus genetic drift as the driving force of the faster differentiation of the Z chromosome (*fast‐Z* effect) and the disproportionate role of the Z chromosome in reproductive isolation (*large‐Z* effect) are still debated. Here, we address this debate in the bird genus *Ficedula* flycatchers based on population‐level whole‐genome sequencing data of six species. Our analysis provides evidence for both faster lineage sorting and reduced gene flow on the Z chromosome than the autosomes. However, these patterns appear to be driven primarily by the increased role of genetic drift on the Z chromosome, rather than an increased rate of adaptive evolution. Genomic scans of selective sweeps and fixed differences in fact suggest a reduced action of positive selection on the Z chromosome.

## 1 INTRODUCTION

The number and genomic distribution of genetic loci that contribute to adaptation and reproductive isolation is a central question in speciation research. Speciation genomic studies across a wide range of taxa have revealed a heterogenous differentiation landscape along the genome, where the heterogeneity is frequently attributed to divergent selection and so‐called barrier loci that build up resistance to gene flow earlier than the genomic background (Feder et al., 2012; Nosil et al., 2009; Via & West, 2008). However, the variability in recombination rate and thus in the intensity of linked selection is also known to contribute to the heterogeneity in differentiation (Nachman & Payseur, 2012; Ravinet et al., 2017; Wolf & Ellegren, 2017). In addition, genomic barriers to gene flow can arise through fixation of different alleles in divergent lineages across multiple loci that result in hybrid incompatibilities when brought into the same genome. These negative epistatic interactions are known as Bateson–Dobzhansky–Muller incompatibilities (BDMIs) and do not need to invoke natural selection, but may be driven entirely by genetic drift (Seehausen et al., 2014).

Sex chromosomes are assumed to make a disproportionally large contribution to hybrid dysfunction and reproductive isolation, commonly referred to as the ‘*large‐X* (or *large‐Z*) effect’, since sex chromosomes more readily expose incompatible genetic loci in hybrids of the heterogametic sex (Coyne, 1984; Presgraves, 2008; Storchová et al., 2010). Furthermore, sex‐linked genetic loci are exposed to selection more efficiently in the heterogametic sex, which increases the efficacy of selection on sex chromosomes (Avery, 1984). Provided that beneficial mutations are on average recessive, this may lead to faster evolution of the X (or Z) chromosome, commonly referred to as the ‘*fast‐X* (or *fast‐Z*) effect’ (Charlesworth et al., 1987). In line with these hypotheses, genomic studies have revealed elevated differentiation levels on sex chromosomes compared to autosomes (Presgraves, 2018). However, elevated differentiation on sex chromosomes does not necessarily reflect a disproportionate contribution of sex chromosomes to adaptation and reproductive isolation (Coyne, 2018; Presgraves, 2018). The reasons for elevated differentiation on sex chromosomes can in fact be manifold.

The sex chromosomes spend unequal times in males and females, and are therefore differently affected by sex‐specific selection mechanisms (Charlesworth et al., 1987; Rice, 1984) and demography (Pool & Nielsen, 2007). Moreover, sex chromosomes and autosomes have different effective population sizes (*N*_e_) (Vicoso & Charlesworth, 2009), mutation rates (Ellegren, 2007; Hedrick, 2007; Kirkpatrick & Hall, 2004) and recombination rates (Hedrick, 2007), which can all contribute to differences in the genomic differentiation landscape among sex chromosomes and autosomes. Since birds have a female heterogametic sex determination system (ZW females and ZZ males), we will in the following discuss these differences from the angle of a ZW sex determination system. Similar arguments apply to an XY sex determination system but with switching the sexes (Irwin, 2018).

Given an equal proportion of reproducing females and males in the population, the Z chromosome versus autosome (Z:A) ratio of *N*_e_ is 3/4 (Vicoso & Charlesworth, 2009), but can range between 9/16 and 9/8 if differences in reproductive variance between sexes are present. These differences in *N*_e_ among the Z chromosome and autosomes will naturally impact the selection–drift balance and for commonly observed Z:A ratios <1 lead to a lower efficacy of selection on the Z chromosome. Indeed, ample evidence suggests that the *fast‐Z* effect in birds is more likely a result of less efficient purging of deleterious mutations due to a higher impact of genetic drift rather than a result of more efficient positive selection in the heterogametic females (Hayes et al., 2020; Mank et al., 2010; Wang et al., 2014). Besides affecting the efficacy of selection, a lower *N*_e_ for the Z chromosome also increases the speed of lineage sorting (Presgraves, 2018; Wolf & Ellegren, 2017), which results in faster genomic differentiation (or higher *F*_ST_) on the Z chromosome than the autosomes. Genomic signatures of a *large‐Z* effect that purely rely on elevated genomic differentiation therefore do not necessarily need to invoke genetic incompatibilities in hybrids that prevent gene flow on the Z chromosome. Moreover, mutation rate is generally found to be higher in males (male‐biased) in birds (Axelsson et al., 2004; Wang et al., 2014), which increases between species genomic differentiation of the Z chromosome relative to autosomes and could be another confounding factor of signatures of *fast‐Z* or *large‐Z* effects. Additionally, since the heteromorphic parts of sex chromosomes only recombine in the homogametic sex, the Z chromosome is observed to show lower rates of recombination compared to autosomes across birds (Wang et al., 2014). A lower recombination rate can result in a reduced efficacy of selection by increasing selective interference between sites (Hill & Robertson, 1966), further impacting the selection–drift balance on sex chromosomes. Additionally, the impact of linked selection is greater when recombination rate is lower, which may manifest in increased genomic differentiation between species (Wolf & Ellegren, 2017). The increased effect of linked selection can also drive a positive relationship between signatures of introgression and recombination rate, where lower levels of introgression coincide with low recombination rate (Martin et al., 2019; Schumer et al., 2018). However, the observation of lower levels of introgression in low recombining regions need not be explained by an increase in genetic incompatibilities in low recombining regions. Instead, selection against gene flow is likely to extend over a wider physical range in low recombining regions. Furthermore, recombination rate is positively correlated with the rate of GC‐biased gene conversion (gBGC), which can confound tests for the strength of direct selection when failing to account for it (Bolívar et al., 2018). Consequently, a thorough investigation of the evidence for *fast‐Z* and *large‐Z* effects must account for the many confounding factors that result from the different properties of the Z chromosome and the autosomes.

**FIGURE 1:**
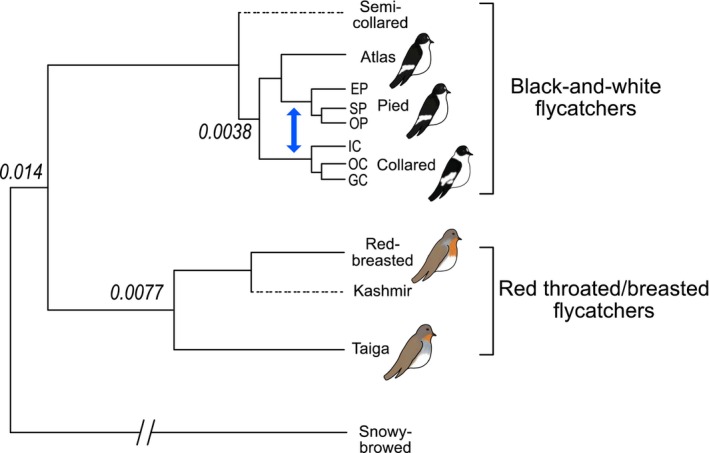
Topological representation of the relationships among the two groups of *Ficedula* flycatcher species studied here. Shown is the species topology of *Ficedula* flycatcher species studied here (represented with solid lines) in addition to remaining species belonging to the two groups not included in the present study (represented with dashed lines). The three node labels represent sequence divergence estimates among species based on *d*_*xy*_ from Chase et al. (2021). The topology for the group of black‐and‐white flycatchers is based on results from Nater et al. (2015) and the topology for the group of red‐throated/breasted flycatchers is based on results of the broader *Ficedula* genus in Moyle et al. (2015). Collared and pied flycatchers are represented by multiple populations, Italian collared flycatcher (IC), Öland collared flycatcher (OC) and Gotland collared flycatcher (GC), Spanish pied flycatcher (EP), Swedish pied flycatcher (SP) and Öland pied flycatcher (OP). The blue arrow depicts the branch on which gene flow was inferred between collared flycatcher and pied flycatcher (see results for the ABBA‐BABA tests).

Here, we evaluate the role of the Z chromosome in adaptation and reproductive isolation in *Ficedula* flycatchers, which are an important avian speciation model (Qvarnström et al., 2010; Sætre & Sæther, 2010). In particular, the naturally hybridizing collared flycatcher (*Ficedula albicollis*) and pied flycatcher (*F*. *hypoleuca*) have been intensively studied in the context of speciation. The two species likely diverged in allopatry and have subsequently come into secondary contact where hybridization occurs (Qvarnström et al., 2010). However, strong reproductive isolation has evolved in the form of both pre‐mating and post‐mating barriers. In sympatry, only a small percentage of matings are heterospecific, and both male and female hybrids that do arise appear to be completely sterile (Ålund et al., 2013; Svedin et al., 2008). The Z chromosome has been proposed a hotspot for reproductive isolation and adaptive speciation in the collared and pied flycatchers (Borge et al., 2005; Sæther et al., 2007; Sætre et al., 2003). However, earlier studies on the role of the Z chromosome have primarily been based on a few markers. More recent genome‐wide approaches have been limited to one type of genomic signature and do not address the role of the Z chromosome in speciation (Nadachowska‐Brzyska et al., 2019; Nater et al., 2015). To provide a comprehensive evaluation of the *fast‐Z* and *large‐Z* effects in *Ficedula* flycatchers, we therefore take advantage of a rich amount of genomic resources comprising of population‐level whole‐genome sequencing data of six species: three populations of collared flycatcher, three populations of pied flycatcher, one population of Atlas flycatcher (*F*. *speculigera*), one population of red‐breasted flycatcher (*F. parva*), one population of taiga flycatcher (*F*. *albicilla*) and one individual of snowy‐browed flycatcher (*F*. *hyperythra*). The collared, pied and atlas flycatchers belong to a group of four black‐and‐white flycatchers (Figure 1) that diverged less than 1 MYA (Nadachowska‐Brzyska et al., 2013) with breeding ranges in Western and Eastern Europe (pied and collared flycatchers, respectively) and North‐west Africa (atlas flycatcher) (Sætre et al., 2001). Phylogenetic analyses have placed the pied and atlas flycatchers as sister species, although hybridization occurs between collared and pied flycatchers in secondary contact zones (Nater et al., 2015). A sister group of the black‐and‐white flycatchers is comprised of three species of red‐breasted/red‐throated flycatchers, the Kashmir flycatcher (*F. subrubra*), the red‐breasted flycatcher and the taiga flycatcher (Figure 1). The Kashmir flycatcher has a geographically limited breeding range in the northwest Himalayas (Bates & Lowther, 1952), while red‐breasted and taiga flycatchers have much broader breeding ranges, spanning from southern Scandinavia into central and eastern Europe and into the Caucasus for red‐breasted flycatcher and from Kamchatka across Siberia and eastern Russia for the taiga flycatcher (Svensson et al., 2005). Although much is known about the ecology of speciation between the collared and pied flycatchers, comparatively little is known regarding the sister group of red‐breasted/red‐throated flycatchers. However, it has been observed that the genomic differentiation between red‐breasted flycatcher and taiga flycatcher is higher than between any of the black‐and‐white flycatchers (Chase et al., 2021; Hung & Zink, 2014), suggesting a deeper divergence time for these species.

With population‐level whole‐genome sequencing data from these birds, we address the following questions: (i) Do the flycatcher species show genomic signatures of an increased rate of evolution on the Z chromosome relative to autosomes (*fast‐Z* effect); (ii) does the Z chromosome show a disproportionate role in reproductive isolation (*large‐Z* effect); and (iii) does adaptation on the Z chromosome play a role in driving these effects.

## 2 MATERIALS AND METHODS

### 2.1 Variant calling and filtering

We compiled a data set of single nucleotide variant (SNV) calls using publicly available whole‐genome sequencing data for 187 samples of four *Ficedula* flycatcher species, including 95 collared flycatcher (Nadachowska‐Brzyska et al., 2021), 11 pied flycatcher (Burri et al., 2015), 15 red‐breasted flycatcher (Chase et al., 2021), 65 taiga flycatcher (Chase et al., 2021) and one individual of an outgroup species, snowy‐browed flycatcher (Burri et al., 2015), for the autosomes and the Z chromosome. Recalibrated BAM files mapped to the collared flycatcher reference genome FicAlb1.5 (Kawakami et al., 2014) were retrieved for all species, with mean mapping percentage of 98.7% for collared flycatcher, 89.9% for pied flycatcher, 91.2% for red‐breasted flycatcher, 97.2% for taiga flycatcher and 72.6% for snowy‐browed flycatcher (Table S1). Subsequently, we performed variant calling of all individuals following the procedure outlined below. Variant calls for autosomal scaffolds were retrieved from Chase and Mugal (2022), and variant calling for Z chromosome scaffolds was performed within the present study following previously described methods (Chase & Mugal, 2022), with additional filtration steps applicable to the Z chromosome. Briefly, genotyping was performed using GATK v.4.1 Haplotype Caller for all individuals separately, followed by joint genotyping with GenotypeGVCFs (McKenna et al., 2010). Genotyping was performed using the flag *–all‐sites* to genotype both polymorphic and monomorphic positions. Genotypes with a sequencing depth below 5× and above 200× were removed, as were autosomal genotypes with genotype quality (GQ) below 30. For the Z chromosome scaffolds, we applied a filter of GQ >30 for male samples and GQ >15 for female samples, since females have only one copy of the Z chromosome in birds. Additionally, we removed any sites with heterozygous genotypes in females after applying genotype filters. Finally, after performing all filters, we removed sites with more than 10% missing data in any of the four species and sites that overlapped with annotated repeats (Suh et al., 2018). Our final data set included 51,424,863 SNVs within a set of 566,724,393 callable sites in total on the autosomes from Chase and Mugal (2022), combined with 2,662,105 SNVs within a set of 30,171,840 callable sites in total on the Z chromosome. Here, SNV calls represent single nucleotide polymorphisms (SNPs) within species and single nucleotide differences among species.

We used an additional data set of SNV calls for introgression tests, with multiple populations of collared flycatcher and pied flycatcher, as well as one population of atlas flycatcher (Burri et al., 2015). These data included 19 Öland collared flycatcher, 20 Italian collared flycatcher, 19 Öland pied flycatcher, 20 Spanish pied flycatcher and 20 Atlas flycatcher. One individual of red‐breasted flycatcher and one individual of snowy‐browed flycatcher were included as outgroups. The SNV calling is described in Burri et al. (2015).

### 2.2 Estimates of the Z/A ratio of effective population size

We computed four estimates of the ratio of effective population size (*N*_e_) on the Z chromosome compared to the autosomes. First, we calculated the ratio of nucleotide diversity π_Z_/π_A_ across the entire assembled autosomes and the Z chromosome. We calculated π for the autosomes and the Z chromosome following the equation:

$$\pi =\frac{\sum_{i=1}^{s}2p_{i}q_{i}}{L}$$

where *p*_i_ and *q*_i_ represent the allele frequencies at site *i* in *s* variable sites. We then obtained the per site measure of π by dividing by the total number of callable sites, *L*, from the vcf containing both monomorphic and polymorphic sites. We estimated π for both the autosomes and the Z chromosome using either all SNPs, or only GC‐conservative SNPs (Strong‐to‐Strong: S‐to‐S and Weak‐to‐Weak: W‐to‐W) to account for GC‐biased gene conversion (gBGC) (Bolívar et al., 2018). In addition, we calculated the ratio of nucleotide diversity π_Z_/π_A_ after masking sites potentially affected by linked selection, which may be stronger on the Z chromosome due to on average lower recombination rate. For this purpose, we masked sites in the reference genome overlapping with both exons (Ensembl version 104; Uebbing et al., 2016) and conserved noncoding elements with a minimum size of 100 bp (CNEs; Craig et al., 2018), and an additional 1 kb flanking region on both sides of the exons and CNEs. To obtain confidence intervals, we randomly resampled variable sites with replacement, for 1000 bootstrap replicates. We refer to these three measures of π_Z_/π_A_ as π_Z_/π_A_ (All sites), π_Z_/π_A_ (GC cons), π_Z_/π_A_ (No LS).

Besides diversity‐based estimates of *N*_e_, we computed historical variation in *N*_e_ separately for the Z chromosome and the autosomes using the Pairwise Sequentially Markovian Coalescent (PSMC) model implemented in the PSMC software (Li & Durbin, 2011). We performed PSMC estimation for one individual of each of the four species (Table S1), which were chosen to minimize differences in mean coverage between sequences as this can bias estimates of *N*_e_ (Nadachowska‐Brzyska et al., 2016). We excluded sites with a read depth below 10 and masked sites with more than twice the average read depth across the genome. Blocks of 100 bp containing more than 20% missing data were excluded. We ran PSMC following (Nadachowska‐Brzyska et al., 2016), and set the input parameters to ‐p ‘4 + 30*2 + 4 + 6 + 10’, ‐t5 and ‐r1. We performed 100 bootstrap replicates by splitting chromosome sequences into segments with ‘splitfa’ and randomly sampling segments with replacement. *N*_e_ estimates were rescaled with the ‘psmc.results’ function from Liu and Hansen (2017) customized by Leroy et al. (2021), using a generation time of 2 years and a mutation rate of 4.6 × 10^−9^ per site per generation (Smeds et al., 2016). We then estimated the ratio of the harmonic mean *N*_e_. For each species, we took the average *N*_e_ estimates from PSMC in 1000‐year discrete time steps from the most recent time up until 1 mya, separately for the Z‐chromosome and the autosomes, and estimated the ratio PSMC_Z/A_. Finally, we estimated 95% confidence intervals based on the harmonic mean *N*_e_ for each of the 100 bootstraps.

For all estimates of *N*_e_ on the Z chromosome compared to the autosomes, either based on genetic diversity or PSMC, we corrected the ratio for the impact of male‐biased mutation rates by dividing all Z chromosome estimates of *N*_e_ by 1.1, following Irwin (2018).

### 2.3 Ancestral sequence reconstruction

We polarized SNPs in the four ingroup *Ficedula* flycatcher species using snowy‐browed flycatcher as an outgroup, which were then used for selective sweep detection (see below). For this purpose, we combined collared and pied flycatcher samples to form a second outgroup to polarize SNVs for red‐breasted and taiga flycatchers, and vice versa. An allele was identified as ancestral when any two of the three groups (snowy‐browed flycatcher, collared and pied flycatcher, or red‐breasted and taiga flycatcher) were fixed for the same allele. Using this approach, we were able to polarize 49,121,805 SNVs on the autosomes and 2,561,370 SNVs on the Z chromosome.

With the polarized sites, we then reconstructed the ancestral sequence from the collared flycatcher reference genome (version FicAlb1.5). We first masked sites that were not genotyped based on the allsites VCF. Then, we masked variable sites that were unable to be polarized, as their ancestral state is equivocal. Finally, we replaced the collared reference allele with the ancestral allele.

### 2.4 Estimates of selection in protein‐coding regions

We estimated selection in protein‐coding sequences on the autosomes and the Z chromosome based on the ratio of non‐synonymous over synonymous nucleotide diversity (π_N_/π_S_), the ratio of non‐synonymous over synonymous nucleotide divergence (*d*_N_/*d*_S_) and the adaptive rate of evolution (ω_a_). We estimated π_N_/π_S_ for all four species by identifying zero‐fold and four‐fold degenerate sites from coding sequences for the ancestral genome reconstruction described above. We then subset the polymorphic sites to only GC‐conservative polymorphisms in order to account for gBGC (Bolívar et al., 2018).

Estimates of *d*_N_/*d*_S_ were obtained for collared flycatcher based on one‐to‐one orthologues between collared flycatcher and zebra finch (*Taeniopygia guttata*), using chicken (*Gallus gallus*) sequences as an outgroup. For this purpose, one‐to‐one orthologues were downloaded from Ensembl version 104. Based on the collared flycatcher reference genome, we identified 7559 genes located on autosomes and 303 genes on the Z‐chromosome. For each gene, we then aligned the orthologous sequences across the three species using PRANK v170427 (Löytynoja, 2014) with help of a guide tree estimated with ClustalW v2.1 (Larkin et al., 2007). To estimate *d*_N_/*d*_S_, we used the software Bio++ v3.0 (Dutheil & Boussau, 2008), which first estimates a gene tree based on maximum likelihood, and then maps substitutions based on stochastic mapping. The gene tree for each gene was estimated using a strand‐symmetric L95 model (Lobry, 1995). We separated substitutions into different categories, to estimate *d*_N_ and *d*_S_ using only GC‐conservative changes to account for gBGC. To average *d*_N_ and *d*_S_ across genes, we weighted the counts for S‐to‐S and W‐to‐W substitutions by the proportion of GCs and ATs and took the sum of the two GC‐conservative substitution types for nonsynonymous and synonymous substitutions.

We estimated the rate of adaptive substitutions, ω_a_, with the software DFE‐alpha (Eyre‐Walker & Keightley, 2009; Keightley & Eyre‐Walker, 2007) using the divergence estimates described above and polymorphism data from all four species separately. Using GC‐conservative sites, we obtained the site frequency spectrum (SFS) for zerofold and fourfold degenerate sites for each species. We then estimated the distribution of fitness effects (DFE) based on the 2‐epoch model of population size change implemented in DFE‐alpha. This resulted in one ω_a_ estimate based on polymorphisms within each species.

We obtained confidence intervals for π_N_/π_S_, *d*_N_/*d*_S_ and ω_a_ by randomly resampling genes with replacement and re‐estimating each statistic for 100 bootstrap replicates.

### 2.5 Population genomics statistics

We estimated *F*_ST_ for the two sister species pairs, collared and pied flycatcher and red‐breasted and taiga flycatcher, for 200‐kb genomic windows along the Z chromosome. Following Chase and Mugal (2022), *F*_ST_ was estimated using VCFtools (Danecek et al., 2011). We identified *F*_ST_ peaks by Z transforming *F*_ST_ values for each chromosome, and applying a Savitzky–Golay filter to the transformed values. Windows with a smoothed Z‐*F*_ST_ value above two were then identified as an *F*_ST_ peak. Estimates for *F*_ST_ along the autosomes were retrieved from Chase and Mugal (2022). To identify branch‐specific signatures of selection, we estimated the population branch statistic (PBS) measure of *F*_ST_, which identifies the contribution to *F*_ST_ from each branch individually. For this purpose, we used taiga flycatcher as an outgroup to estimate PBS in collared and pied flycatchers, and used collared flycatcher as an outgroup to estimate PBS in red‐breasted and taiga flycatchers. To identify outlier PBS windows, we applied the same Z‐score threshold as described above for *F*_ST_.

In addition to estimating *F*_ST_, we performed a selective sweep scan along the Z chromosome to look for signatures of positive selection. We used the program SweepFinder2 (DeGiorgio et al., 2016) to implement the composite likelihood ratio (CLR) test (Nielsen et al., 2005), using the polarized SNP data for all four species individually. SweepFinder2 was run using the ‐ug option with a pre‐computed background SFS for the Z chromosome for each species and a user‐defined grid with the location for each variant. Sites were first filtered to remove positions fixed for the ancestral allele within each species. We determined the significance threshold for the CLR test based on simulations in SLiM 3 (Haller & Messer, 2019). We simulated background selection occurring across an approximately 21 Mbp chromosome, based on gene density and recombination rate estimates from the collared flycatcher. The significance threshold based on these simulations is 46.25. We merged adjacent sites with significant CLR values into a single sweep region and removed sweeps that contained only one position or that had a site density less than 1 bp/1 kb, and then obtained the presence/absence of selective sweeps in 200‐kb windows. Estimates for selective sweeps along the autosomes were retrieved from Chase and Mugal (2022).

### 2.6 Identification of fixed differences and shared polymorphisms

We identified sites that showed fixed differences between collared and pied flycatcher and between red‐breasted and taiga flycatcher, as well as sites that displayed shared polymorphisms between the two species comparisons. We overlapped fixed differences with different functional categories and compared the relative proportions of fixed differences in each category on the Z chromosome compared to the autosomes. Functional categories included intergenic regions, intronic regions, conserved non‐coding elements (CNEs) (Craig et al., 2018), untranslated regions (UTRs), fourfold degenerate sites and zerofold degenerate sites, where the latter three are based on the collared flycatcher annotation (Ensembl v. 104; Uebbing et al., 2016). Additionally, we examined whether any nonsynonymous fixed differences overlapped with a signature of selective sweeps in either of the two species compared.

### 2.7 Estimates of gene flow

Collared and pied flycatcher have partially overlapping breeding ranges, and produce hybrid offspring in those contact zones. F1 hybrids are generally found to be sterile (Ålund et al., 2013; Svedin et al., 2008). Nevertheless, previous demographic modelling suggests a recent history of gene flow between the two species (Nadachowska‐Brzyska et al., 2013; Nater et al., 2015). Less is known about the red‐breasted and the taiga flycatcher (Hung & Zink, 2014; Svensson et al., 2005). For this reason and due to limited data availability of relevant reference species for the red‐breasted and the taiga flycatcher, we here focus on gene flow between the collared and pied flycatcher. Specifically, we compared rates of gene flow between collared and pied flycatcher on the autosomes compared to the Z chromosome using Patterson's D statistic (Green et al., 2010). This test takes a four‐taxon comparison, (P1, P2, P3, O), where O represents an outgroup, and can test for gene flow between population 1 (P1) and population 3 (P3) or between population 2 (P2) and P3. We estimated Patterson's D for multiple population comparisons, which allowed us to investigate at what stage during the divergence of collared and pied flycatchers gene flow occurred. First, we set Atlas flycatcher as P1 and set Öland pied flycatcher and collared flycatcher as P2 and P3, respectively, and performed a separate test with Spanish pied flycatcher and Italian collared flycatcher as P2 and P3. Second, we used Spanish pied flycatcher as P1, Öland pied flycatcher as P2 and Öland collared flycatcher as P3, to determine whether there was greater evidence for gene flow between Öland populations. Third, we performed the test for Italian collared flycatcher as P1, Öland collared flycatcher as P2 and Öland pied flycatcher as P3. For each comparison, we used red‐breasted flycatcher and snowy‐browed flycatcher as outgroups. Polarized polymorphism data for all four species were obtained from previously published work (Burri et al., 2015).

We estimated Patterson's D for autosomes and the Z chromosome separately, using the derived allele frequencies (*p*) in the three in‐group species with the formula:

$$D=\frac{\sum \left(1-p_{1}\right)\times p_{2}\times p_{3}-\sum p_{1}\times \left(1-p_{2}\right)\times p_{3}\;}{\sum \left(1-p_{1}\right)\times p_{2}\times p_{3}+\sum p_{1}\times \left(1-p_{2}\right)\times p_{3}\;}$$

To test whether the estimates of Patterson's D calculated were significantly different from zero, and thus showing a signature of gene flow, we performed jackknife resampling, removing blocks of 200 kb to estimate standard error and a Z‐score for both the autosomes and the Z chromosome.

In addition to autosomal and Z chromosome average estimates of gene flow, we performed a window‐based analysis to obtain local estimates of gene flow across the genome. We estimated the *f*_*d*_ statistic (Martin et al., 2015), which was developed to account for the high variance observed in the D‐statistic in small genomic regions. We estimated this statistic only for the species comparisons that showed statistically significant estimates of gene flow on both autosomes and the Z‐chromosome, since the *f*_*d*_ statistic is designed to detect gene flow between P2 and P3 and is not biologically meaningful when negative. We then identified genomic windows showing significantly reduced gene flow by first applying a smoothing algorithm to the window‐based estimates of *f*_*d*_ for each chromosome, and then Z‐transforming the smoothed estimates using the genome‐wide mean and standard deviation. Windows with *z*‐scores of −2 or lower were identified as significant outliers.

### 2.8 Statistical analysis

All statistical analysis was performed in R version 4.0.3 (R Core Team, 2020).

## 3 RESULTS

### 3.1 Z/A ratio of effective population size in four *Ficedula* flycatcher species

**FIGURE 2:**
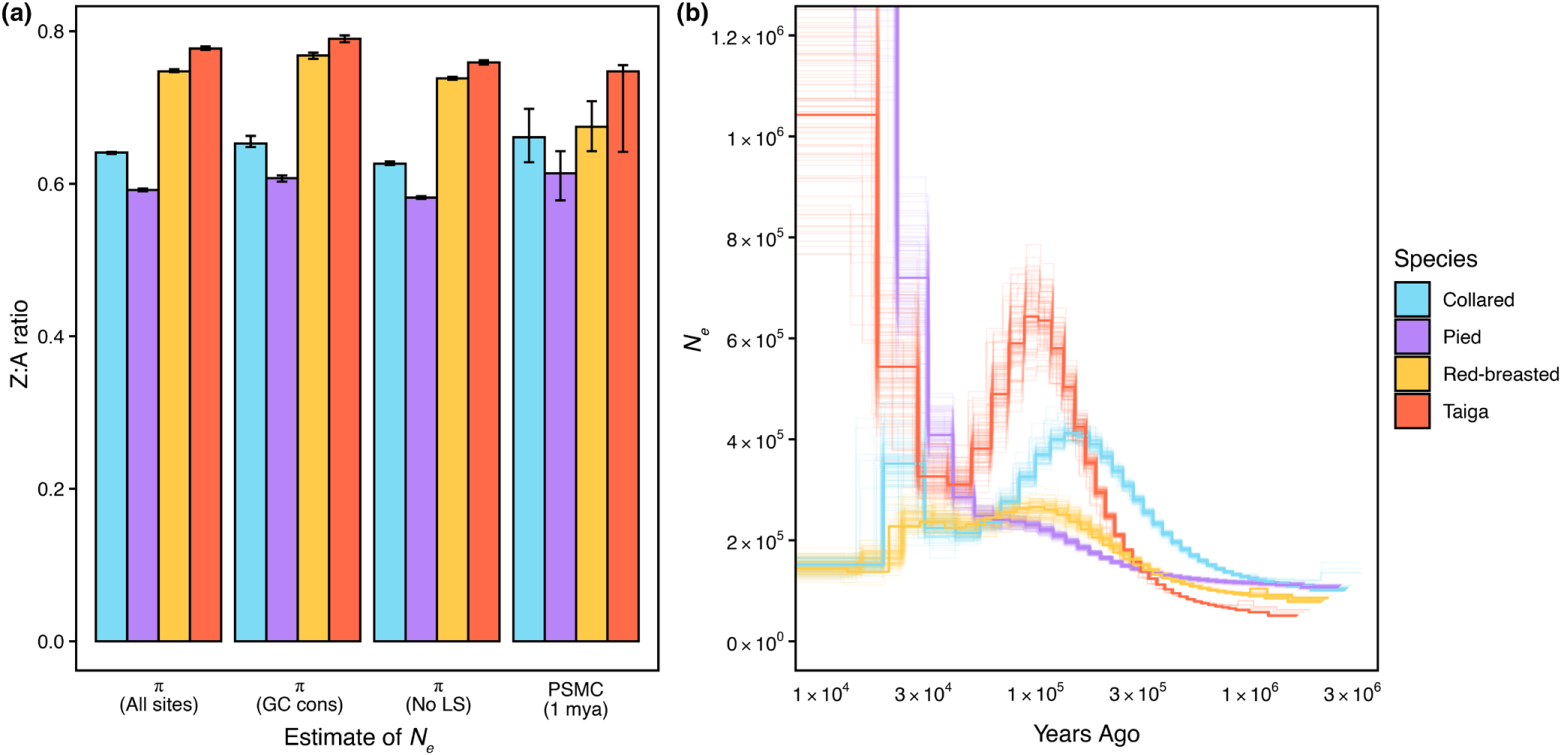
Effective population size (*N*_e_) on the autosomes and the Z chromosome. (a) The ratios of different estimates of *N*_e_ for all four species on the Z chromosome versus the autosomes (Z:A ratio). Estimates of *N*_e_ are based on nucleotide diversity (π) using all SNPs (All sites), only GC‐conservative SNPs (GC cons), π corrected for linked selection by masking protein‐coding sequences and conserved non‐coding elements and their 1 kb flanking regions (No LS), and estimates based on PSMC historical population size for the last 1Mya. All estimates are corrected for male‐biased mutation rate. See Tables S2 and S3 for respective estimates for the Z chromosome and autosomes. (b) Historical changes in population size for all four species estimated on the autosomes. The bold line represents the genome‐wide estimate; bootstrap replicates are shown in lighter colour. See Figure S1 for estimates on the Z chromosome.

For the four focal flycatcher species, we estimated the ratio of *N*_e_ on the Z chromosome compared to autosomes with four different measures, π_Z_/π_A_ (All sites), π_Z_/π_A_ (GC cons), π_Z_/π_A_ (No LS) and PSMC_Z/A_, which were all largely consistent within species (Figure 2a; Tables S2 and S3). Collared flycatcher and pied flycatcher showed a ratio clearly below 0.75, while red‐breasted flycatcher and taiga flycatcher showed larger values close to 0.75 for all four estimates (Figure 2a). The consistency among π_Z_/π_A_ estimates suggests that differences in the strength of gBGC and/or linked selection between the Z chromosome and the autosomes do not show an impact on the Z:A ratio of *N*_e_ in *Ficedula* flycatchers. The Z:A ratio below 0.75 in both collared flycatcher and pied flycatcher could not be explained by similar demographic histories based on PSMC (Figure 2b). However, a higher variance of reproductive success in males observed in the partly polygynous collared flycatchers and pied flycatchers (Storchová & Hořák, 2018) is in good agreement with the observed reduction Z:A ratios below 0.75 in these two species. In comparison, red‐breasted flycatchers are shown to be purely monogamous (Storchová & Hořák, 2018; data not available for taiga flycatchers) and show Z:A ratios close to 0.75 as expected under equal variance in reproductive success between the sexes.

### 3.2 No evidence that adaptation drives the *fast‐Z* effect in *Ficedula* flycatchers

Figure 2a illustrates that *N*_e_ is smaller on the Z chromosome than the autosomes in all four species. We therefore examined if these differences in *N*_e_, and hence the strength of genetic drift, and/or the hemizygote state of the Z chromosome in females influence the efficacy of natural selection on protein‐coding sequences. We observed that π_N_/π_S_ was higher on the Z chromosome compared to the autosomes for all four species (Table 1). Similarly, branch‐specific *d*_N_/*d*_S_ estimates for the flycatcher lineage after the split from zebra finch was higher on the Z chromosome, which is consistent with a *fast‐Z* effect in *Ficedula* flycatchers. To assess if elevated *d*_N_/*d*_S_ is a result of relaxed purifying selection or stronger positive selection on the Z chromosome, we estimated the adaptive substitution rate ω_a_. We used polymorphism data of each of the four species separately to estimate the distribution of fitness effects, which provides information on the influence of demography on ω_a_ estimates. This revealed that estimates of ω_a_ were lower on the Z chromosome compared to the autosomes for pied flycatcher and taiga flycatcher (Table 1), which both show a recent increase in population size (Figure 2b). Collared flycatcher and red‐breasted flycatcher, which both show less fluctuation in recent population size (Figure 2b), showed higher or not significantly different estimates on the Z chromosome compared to the autosomes. These differences in ω_a_ estimates among species are solely governed by differences in the SFS among species, which is also apparent in differences in π_N_/π_S_ estimates. The impact of demography on estimates of ω_a_ therefore makes it difficult to assess the role of adaptation in the *fast‐Z* effect.

**TABLE 1:** Estimates of selection on coding sequences for autosomes and the Z chromosome.

	Collared	Pied	Red‐breasted	Taiga
π_N_/π_S_ A	0.174 [0.159;0.186]	0.186 [0.166;0.209]	0.164 [0.148;0.175]	0.169 [0.155;0.181]
π_N_/π_S_ Z	0.183 [0.116;0.262]	0.344 [0.163;0.653]	0.194 [0.139;0.284]	0.229 [0.166;0.309]
*d*_N_/*d*_S_ A	0.171 [0.164;0.177]			
*d*_N_/*d*_S_ Z	0.198 [0.166;0.233]			
ω_a_ A	0.0592 [0.0434;0.0767]	0.0422 [0.0199;0.0668]	0.0451 [0.0259;0.0622]	0.0900 [0.0771;0.100]
ω_a_ Z	0.112 [0.0509;0.187]	0.0182 [−0.0896;0.107]	0.0474 [−0.0533;0.129]	0.0594 [−0.0274;0.104]

*Note*: Shown are estimates of π_N_/π_S_, *d*_N_/*d*_S_ and ω_a_ separately for genes on the autosomes and on the Z chromosome. All estimates are based on GC‐conservative sites only. See Table S4 for estimates based on all sites.

**FIGURE 3:**
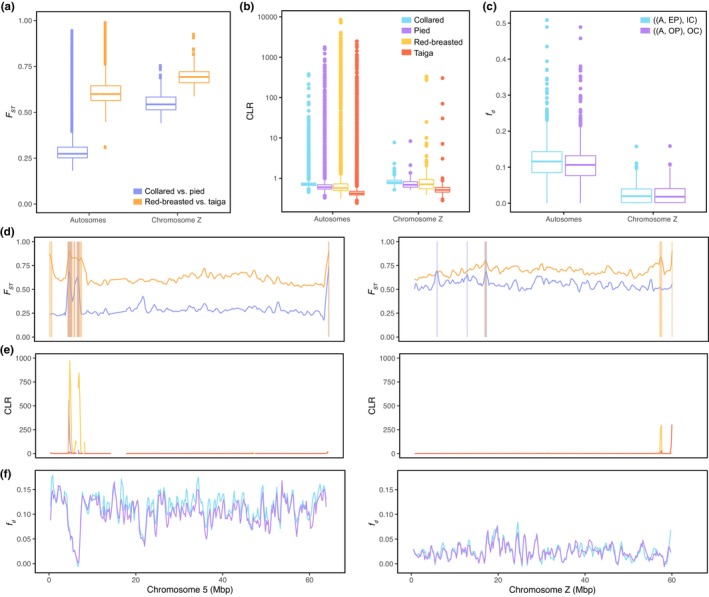
Signatures of linked selection and gene flow on the Z chromosome compared to autosomes. (a) The distributions of window‐based estimates of *F*_ST_ between collared and pied flycatchers (purple) and red‐breasted and taiga flycatchers (orange) separately for autosomes and the Z chromosome. (b) Window‐based CLR estimates for the four species in log‐scale. (c) Window‐based estimates of *f*_*d*_ for gene flow estimates between Spanish pied flycatcher and Italian collared flycatcher (blue) and between Öland pied flycatcher and Öland collared flycatcher (purple) using atlas flycatcher as a reference. (d) Measures of *F*_ST_ estimated in 200‐kb genomic windows for chromosome 5 (left) and the Z chromosome (right). Shaded rectangles demonstrate the locations of significant *F*_ST_ peaks in both species comparisons. (e) CLR estimates for collared (blue), pied (purple), red‐breasted (yellow) and taiga (red) flycatcher on both chromosomes. (f) Window‐based estimates of *f*_*d*_ for gene flow estimates between Spanish pied flycatcher and Italian collared flycatcher (blue) and between Öland pied flycatcher and Öland collared flycatcher (purple). See Figure S2 for estimates of *F*_ST_ for all chromosomes, Figure S3 for estimates of CLR for all chromosomes and Figure S4 for estimates of *f*_*d*_ along all chromosomes.

To complement the analysis based on estimates of selection on protein‐coding sequences, we estimated window‐based *F*_ST_ on the Z chromosome in 200‐kb windows for the two sister species comparisons: collared and pied flycatchers and red‐breasted and taiga flycatchers (Figure 3a,d). For both comparisons, *F*_ST_ was notably higher on the Z chromosome than on the autosomes, with Z chromosome average *F*_ST_ 0.55 versus 0.29 for the autosomes between collared and pied flycatcher and 0.70 versus 0.62 between red‐breasted and taiga flycatcher. We found that *F*_ST_ peaks occurred more frequently on autosomes for red‐breasted and taiga flycatcher (Table 2), while there was no significant difference for collared and pied flycatcher. Thus, the higher *F*_ST_ levels on the Z chromosome appeared to be a chromosome‐wide effect resulting from faster lineage sorting rather than more prevalent signatures of *F*_ST_ peaks. Since selective sweeps have been found to play a role in shaping *F*_ST_ peaks in *Ficedula* flycatchers (Chase et al., 2021), the low prevalence of *F*_ST_ peaks on the Z chromosome in fact suggests that linked positive selection is not more common on the Z chromosome compared to the autosomes.

**TABLE 2:** Number of *F*_ST_ peaks on autosomes compared to the Z chromosome.

	Autosome	Z chromosome	Odds ratio	*p*‐Value
Coll/pied *F*_ST_ peak	179	5	2.2	.10
Coll/pied no *F*_ST_ peak	4517	278		
Red‐breasted/taiga *F*_ST_ peak	247	6	2.6	.017
Red‐breasted/taiga no *F*_ST_ peak	4449	277		

*Note*: Shown are the number of 200‐kb windows overlapping with an *F*_ST_ peak or not for both species comparisons, for both the autosomes and the Z chromosome. Odds ratio and *P*‐value indicate the significance level of the overrepresentation on the autosomes based on Fisher exact tests.

To corroborate this finding, we compared selective sweep scans in each species on the autosomes and Z chromosome (Figure 3b,e), as well as estimating the population branch statistic (PBS) for each species. Consistent with observations for *F*_ST_ peaks, red‐breasted and taiga flycatchers showed a greater proportion of selective sweeps on the autosomes than the Z chromosome, while collared flycatcher and pied flycatchers showed no significant difference between the autosomes and the Z chromosome (Table 3). In addition, we found that selective sweep signatures on the Z chromosome overlapped on average with a lower fraction of nonsynonymous fixed differences compared to the autosomes in all species (Table S5). PBS results also provided no evidence for increased positive selection on the Z chromosome, with all species showing no significant difference in PBS outliers on the autosomes compared to the Z chromosome (Table S6).

**TABLE 3:** Prevalence of selective sweeps on autosomes compared to the Z chromosome.

	Autosome	Z chromosome	Odds ratio	*p*‐Value
Collared sweep	59	1	4.2	.18
Collared no sweep	4715	335		
Pied sweep	137	6	1.6	.30
Pied no sweep	4588	327		
Red‐breasted sweep	248	9	2.0	.039
Red‐breasted no sweep	4512	332		
Taiga sweep	223	5	3.3	.0027
Taiga no sweep	4623	341		

*Note*: Shown are the number of 200‐kb windows overlapping with a significant selective sweep signature on the autosomes and the Z chromosome compared to the number of 200‐kb windows not overlapping with a selective sweep. Odds ratio and *p*‐value indicate the significance level of the overrepresentation on the autosomes based on Fisher exact tests.

**FIGURE 4:**
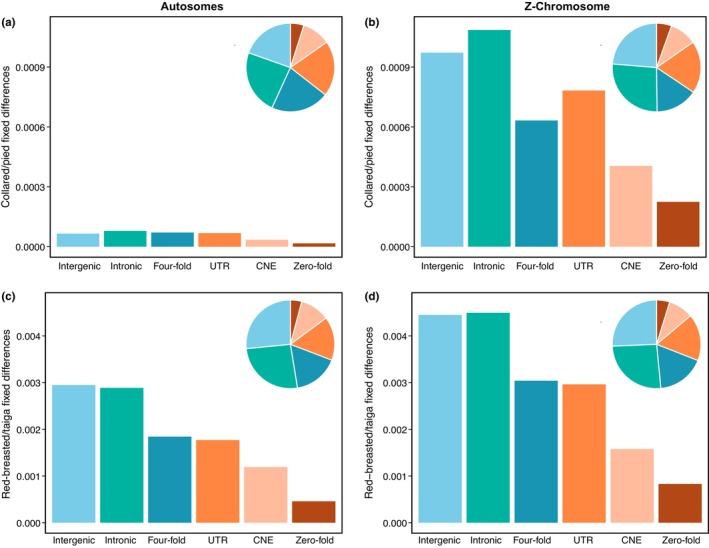
Functional overlap of fixed differences on the Z chromosome versus the autosomes. (a, b) The densities of fixed differences in different functional regions between collared and pied flycatcher on the autosomes and the Z chromosome, respectively. (c, d) The same between red‐breasted and taiga flycatcher. In all four panels, fixed differences are grouped into the following functional categories: intergenic, intronic and untranslated regions (UTRs), conserved noncoding elements (CNEs), four‐fold degenerate sites and zero‐fold degenerate sites. Colours correspond to whether differences are suggested to be evolving under selective constraint (orange shade) or neutrally (blue shade). The pie chart insets show the proportion of fixed differences in each functional category.

We next computed the density of fixed differences between both collared and pied flycatcher and red‐breasted and taiga flycatcher in six different functional regions of the genome, that is, intergenic and intronic regions, UTRs, CNEs, as well as four‐fold and zero‐fold degenerate sites. For both species pairs, we observed the highest density of fixed differences in introns and intergenic regions on both the autosomes and the Z chromosome, followed by four‐fold degenerate sites and UTRs (Figure 4). Both CNEs and zero‐fold degenerate sites showed the lowest density of fixed differences for both species pairs and chromosome types (Figure 4). Overall, there was no observable increase of fixed differences in functional categories potentially evolving under selective constraint (UTRs, CNEs and zero‐fold degenerate sites) versus potentially neutrally evolving categories (intronic regions, intergenic regions and four‐fold degenerate sites) for the Z chromosome versus autosomes (Table S7). These results suggest that the increase in fixed differences on the Z chromosome is driven primarily by faster lineage‐sorting rather than positive selection. In line with this conclusion, we also find fewer shared polymorphisms between the two species pairs on the Z chromosome compared to autosomes (Table S8).

### 3.3 Reduced signatures of introgression on the Z chromosome

We performed ABBA‐BABA tests to detect signatures of introgression for several combinations of populations of collared flycatcher and pied flycatcher. We found significant evidence for a history of gene flow between collared flycatcher and pied flycatcher for Öland populations of both species and for Italian collared flycatcher and Spanish pied flycatcher (Table 4), using Atlas flycatcher as a reference species. In both tests, the Z chromosome showed a lower value of the D‐statistic compared to the autosomes (Table 4), suggesting there has been a relative reduction in gene flow on the Z chromosome. Between the sympatric Öland populations of collared flycatcher and pied flycatcher, using either Italian collared flycatcher or Spanish pied flycatcher as reference species, we found no significant evidence of gene flow on the Z chromosome, and a significant but small effect size of gene flow on the autosomes (Table 4). Taken together, these D‐statistic estimates point to a more ancient history of gene flow between collared flycatcher and pied flycatcher, which occurred before the divergence of the different collared flycatcher and pied flycatcher populations considered here, and that gene flow is lower on the Z chromosome compared to the autosomes.

**TABLE 4:** Signatures of gene flow on the autosomes compared to the Z chromosome.

Species	Chromosome	D‐statistic (±SE)	Z‐score	*p*‐Value
((A, OP), OC)	Autosomes	0.18 (±0.0012)	145	<10^−12^
((A, OP), OC)	Chromosome Z	0.10 (±0.0089)	11	<10^−12^
((A, EP), IC)	Autosomes	0.18 (±0.0012)	145	<10^−12^
((A, EP), IC)	Chromosome Z	0.099 (±0.0086)	12	<10^−12^
((EP, OP), OC)	Autosomes	0.0018 (±0.00042)	4.3	1.7 × 10^−5^
((EP, OP), OC)	Chromosome Z	−0.0022 (±0.0060)	−0.36	.72
((IC, OC), OP)	Autosomes	−0.0015 (±0.00040)	−3.7	2.2 × 10^−4^
((IC, OC), OP)	Chromosome Z	0.00027 (±0.0037)	0.072	.94

*Note*: Shown are results from ABBA‐BABA tests on the autosomes and Z chromosome for five different population/species comparisons. The populations/species are listed in the format ((P1, P2), P3), where P1 and P2 represent sister populations/species, and where we are testing for evidence of gene flow between either P1 or P2 with P3. Populations/species included in the tests are Atlas flycatcher (A), Öland pied flycatcher (OP), Spanish pied flycatcher (EP), Öland collared flycatcher (OC) and Italian collared flycatcher (IC). *p*‐Values were estimated by block jackknife resampling, removing 200‐kb windows.

We next estimated window‐based signatures of gene flow using the *f*_*d*_ statistic for the two comparisons that showed signatures of gene flow on both autosomes and the Z chromosome. Consistent with genome‐wide ABBA‐BABA tests, this revealed that, in both comparisons, the distribution of *f*_*d*_ values on the Z chromosome was lower than on the autosomes (Figure 3c,f). This observation was apparent for all autosomal windows combined, and for windows separately for each chromosome, with the exception of some microchromosomes with few data points (Figure S4). We identified many windows on the Z chromosome showing significantly reduced estimates of *f*_*d*_ compared to the genome‐wide average, and we found that the Z chromosome was enriched for these windows (Table S9).

Since the Z chromosome has on average lower rates of recombination compared to autosomes (Kawakami et al., 2014), the reduction in recombination rate alone could potentially explain the reduced signatures of gene flow we detect on the Z chromosome (Martin et al., 2019; Schumer et al., 2018). We observed there was a significant relationship between recombination rate estimated in collared flycatcher (Kawakami et al., 2014) and the *f*_*d*_ statistic for both comparisons of populations (Öland collared flycatcher and pied flycatcher: *R* ^2^ = .020, *p*‐value = 1.6 × 10^−13^; Italian collared flycatcher and Spanish pied flycatcher: *R* ^2^ = 0.019, *p*‐value = 9.0 × 10^−13^); however, the low *R* ^2^ demonstrates that recombination rate explains little of the genome‐wide variation in *f*_*d*_.

## 4 DISCUSSION

Our analysis of the Z:A ratio of effective population size (*N*_e_) reveals that collared flycatcher and pied flycatcher show a relatively lower *N*_e_ on the Z chromosome than red‐breasted flycatcher and taiga flycatcher. The Z:A ratio of *N*_e_ was significantly lower than 0.75 in the two black‐and‐white flycatchers, while red‐breasted flycatcher and taiga flycatcher showed a ratio close to the expectation of 0.75 for equal sex ratios. These differences relate very well to the mating behaviours reported for different *Ficedula* flycatchers (Storchová & Hořák, 2018), where a greater reproductive variance in males is expected to reduce the Z:A ratio of *N*_e_ (Vicoso & Charlesworth, 2009). Collared flycatchers and pied flycatchers are partly polygynous, while red‐breasted flycatchers are purely monogamous. Data on mating behaviours for the taiga flycatcher are not available, but might be monogamous given their close relationship with red‐breasted flycatchers. Differences in the strength of linked selection among the Z chromosome and autosomes appear not to show any strong influence on the Z:A ratio of *N*_e_ in the *Ficedula* flycatcher lineage. The lack of a strong impact of linked selection on the Z:A ratio of *N*_e_ suggests caution in the presumption that linked selection might explain low values of Z:A diversity observed in birds (Irwin, 2018), which is further supported by our observation that selective sweep signatures are not more pronounced on the Z chromosome. Also, differences in the demographic history between the four species did not correlate with observed differences in the Z:A ratio of *N*_e_ among species. It therefore appears that life‐history traits and mating behaviour are the strongest predictors of differences in the Z:A ratio of *N*_e_ among *Ficedula* flycatchers.

Despite the observed differences in the Z:A ratio of *N*_e_ among species, *N*_e_ is clearly smaller on the Z chromosome than the autosomes for all four *Ficedula* flycatchers. The stronger impact of genetic drift on the Z chromosome than the autosomes therefore needs to be considered in the evaluation of the driving forces of the *fast‐Z* and *large‐Z* effects. Indeed, macro‐ and micro‐evolutionary signatures of natural selection suggest that genetic drift rather than adaptation explains the *fast‐Z* effect in *Ficedula* flycatchers, which is in line with previous observations in birds (Hayes et al., 2020; Mank et al., 2010; Wang et al., 2014). We find evidence for reduced purifying selection on the Z chromosome, but no evidence for a stronger signature of positive selection on the Z chromosome than the autosomes. On the contrary, if anything, the signature of positive selection appears to be weaker on the Z chromosome than the autosomes. While divergent demographic history results in inconsistent patterns of the rate of adaptive evolution between the Z chromosome and the autosomes among species, comparison of the prevalence of selective sweep signatures provides a clearer picture. For collared flycatcher and pied flycatcher, no significant difference in the prevalence of selective sweeps could be found between the Z chromosome and the autosomes. For red‐breasted flycatcher and taiga flycatcher, selective sweep signatures were clearly less prevalent on the Z chromosome than the autosomes. It is, however, worth noting that if genetic drift primarily drives the *fast‐Z* effect in the *Ficedula* flycatchers, we might expect to observe a stronger *fast‐Z* effect in the collared and pied flycatcher, where the ratio of Z:A *N*_e_ is even lower than 0.75. Our evidence for a stronger effect in these species is somewhat inconclusive. The Z chromosome shows a greater relative increase in *F*_ST_ and the number of fixed differences compared to the autosomes than is observed in red‐breasted and taiga flycatchers, consistent with a greater *fast‐Z* effect. However, this result can also be driven by the comparatively lower divergence time between collared and pied flycatchers than between red‐breasted and taiga flycatchers. Comparisons of the π_N_/π_S_ ratio between the Z chromosome and the autosomes among species are strongly influenced by their recent demographic histories, and are therefore not conclusive. Thus, while genetic drift in general appears to drive the *fast‐Z* effect in all four species, the impact of the variation in the ratio of Z:A *N*_e_ among the species on the extent of the *fast‐Z* effect would require comparisons across a larger data set of species pairs in order to control for confounding factors.

Despite the lack of stronger positive selection on the Z chromosome, we observe evidence for reduced gene flow on the Z chromosome compared to the autosomes. Thus, our results support the hypothesis that the *large‐Z* effect does not necessarily need to invoke positive or divergent selection. The reduction in gene flow appears to be a chromosome‐wide effect rather than limited to narrow barrier loci, which is in good agreement with the chromosome‐wide effect of genetic drift and the chromosome‐wide elevated differentiation. A chromosome‐wide mechanism can further explain the presence of genomic signatures of a *large‐Z* effect despite a lack of ‘active’ differential introgression on the Z chromosome (Hogner et al., 2012). Specifically, the relative reduction in *N*_e_ on the Z chromosome compared to the autosomes leads to faster lineage sorting and elevated *F*_ST_ on the entire Z chromosome, where the latter is frequently perceived as evidence for *fast‐Z* and *large‐Z* effects (Irwin, 2018; Presgraves, 2018). However, even though we do not find signatures of adaptation on the Z chromosome, our analysis does not exclude the possibility that Z‐linked loci could play an important role for hybrid incompatibilities and reproductive isolation. The accelerated differentiation of the Z chromosome could potentially lead to an accelerated accumulation of incompatibilities between the Z chromosome and interacting loci on the autosomes. Thus, both scenarios are possible; the reduction in gene flow due to increased hybrid incompatibilities may drive the apparent increased lineage sorting on the Z chromosome, or the increased lineage sorting may contribute to the faster evolution of genetic incompatibilities. Ultimately, the reduction in gene flow and increased lineage sorting due to lower *N*_e_ need not act sequentially, and both effects may accentuate the other. Within the collared and pied flycatchers, it has been observed that hybrid male sterility is associated with interacting genes on both the Z chromosome and the autosomes (Segami et al., 2022). It is therefore tempting to speculate that faster lineage sorting on the Z chromosome may have led to the fixation of incompatibilities, which in turn triggered a snowball effect of selective sweeps on the autosomes for compensatory mutations within a species. The observed overrepresentation of interacting autosomal and Z‐linked genes involved in meiosis with fixed differences between the two species (Segami et al., 2022) is in good agreement with such a scenario.

## AUTHOR CONTRIBUTIONS

MAC and CFM designed and performed the research study. MAC conducted data analyses. MV contributed to the data analyses. CFM supervised the study. MAC and CFM wrote the manuscript. All authors critically read and approved the final version of the manuscript.

## CONFLICT OF INTEREST STATEMENT

The authors declare no conflict of interest.

## Supporting information


Appendix S1.

## Data Availability

Sequencing data for all samples are available at the EMBL‐EBI European Nucleotide Archive (ENA; http://www.ebi.ac.uk/ena) with the following accession numbers: PRJEB43825 (taiga and red‐breasted flycatchers), PRJEB22864 (collared flycatcher) and PRJEB7359 (pied and snowy‐browed flycatchers). VCF files generated for the present study are available at Dryad (Chase et al., 2023), doi: 10.5061/dryad.2jm63xswp. Scripts used for analysis are publicly available on GitHub, https://github.com/madeline‐chase/flycatcher_z_chr.
