# Supplementary material for: Evidence that genetic drift not adaptation drives fast‐Z and large‐Z effects in Ficedula flycatchers
Source: Mol Ecol. 2024 Jan 9;34(18):e17262. doi: 10.1111/mec.17262 (PMC12421497; doi:10.1111/mec.17262)
Supplement: Supplementary file 1 — Appendix S1. [file MEC-34-e17262-s001.pdf]

SUPPLEMENTARY MATERIAL

Evidence that genetic drift not adaptation drives *fast-Z* and *large-Z* effects in *Ficedula* flycatchers.

Madeline A. Chase, Maurine Vilcot, and Carina F. Mugal

SUPPLEMENTAL TABLES

Table S1: Sample information of individuals included in variant calling

Table S2: Estimates of diversity and PSMC historical population size on autosomes and the Z-chromosome

Table S3: Estimates of diversity on autosomes and the Z-chromosome for GC-conservative polymorphisms

Table S4: Estimates of selection on protein-coding sequences for autosomes and the Z-chromosome

Table S5: Number of nonsynonymous fixed differences overlapping with selective sweeps

Table S6: Number of outlier PBS peaks on autosomes compared to the Z-chromosome

Table S7: Density of fixed differences in neutral regions and functionally constrained regions on the autosomes vs. the Z-chromosome

Table S8: The number of shared SNPs on the autosomes vs. the Z-chromosome for both species comparisons

Table S9: The number of outlier f_d windows on the autosomes vs. the Z-chromosome

SUPPLEMENTAL FIGURES

Figure S1: Historical changes in population size for all four species estimated on the Z-chromosome

Figure S2: Distributions of F_{ST} estimates across chromosomes

Figure S3: Distributions of CLR estimates across chromosomes

Figure S4: Distributions of f_d estimates across chromosomes

Table S1: Sample information of individuals included in variant calling. Shown are the sample IDs, species, mapping percentage, mean sequencing depth, and sex for each individual included in the variant calling. Asterisks (*) denote the individual chosen for each of the four ingroup species for the PSMC analysis.

Sample	Species	Mapping percent	Mean depth	Sex
Sample_93F24	Collared	98.45	38.737	Female
Sample_93F26	Collared	98.42	41.5193	Female
Sample_93F30	Collared	98.43	43.7559	Female
Sample_93F32	Collared	98.54	39.7529	Female
Sample_93F34	Collared	98.53	44.5932	Female
Sample_93F35	Collared	98.47	36.591	Female
Sample_93F42	Collared	98.51	43.5583	Female
Sample_93F44	Collared	98.55	42.9247	Female
Sample_93F45	Collared	98.64	52.5206	Female
Sample_93F47	Collared	98.56	32.7092	Female
Sample_93F54	Collared	98.54	46.2067	Female
Sample_93F56	Collared	98.62	37.6966	Female
Sample_93F59	Collared	98.67	45.4955	Female
Sample_93F74	Collared	98.63	43.6838	Female
Sample_93F75	Collared	98.63	45.88	Female
Sample_93F77	Collared	98.59	35.7116	Female
Sample_93F82	Collared	98.66	34.5523	Female
Sample_93F88	Collared	98.64	37.909	Female
Sample_93F89	Collared	98.61	45.8239	Female
Sample_93F90	Collared	98.56	42.6604	Female
Sample_93F92	Collared	98.48	43.632	Female
Sample_93F93	Collared	98.66	37.7044	Female
Sample_93F94	Collared	98.33	42.9574	Female
Sample_93M25	Collared	98.8	46.5928	Male
Sample_93M27	Collared	98.62	33.6413	Male
Sample_93M28	Collared	98.64	37.8529	Male
Sample_93M29	Collared	98.66	40.1815	Male
Sample_93M36	Collared	98.72	37.18	Male
Sample_93M38	Collared	98.67	41.6555	Male
Sample_93M39	Collared	98.73	39.6918	Male
Sample_93M40	Collared	98.76	43.0164	Male
Sample_93M41	Collared	98.81	40.0335	Male
Sample_93M46	Collared	98.81	46.0985	Male
Sample_93M53	Collared	98.74	35.7277	Male
Sample_93M55	Collared	98.72	40.2893	Male
Sample_93M58	Collared	98.82	41.0307	Male
Sample_93M71	Collared	98.79	38.8732	Male

Sample_93M72	Collared	98.77	36.3432	Male
Sample_93M73	Collared	98.8	39.2506	Male
Sample_93M78	Collared	98.89	40.2156	Male
Sample_93M79	Collared	98.83	41.4265	Male
Sample_93M80	Collared	98.72	34.8153	Male
Sample_93M81	Collared	98.75	52.6582	Male
Sample_93M83	Collared	98.67	33.3957	Male
Sample_93M84	Collared	98.8	36.8297	Male
Sample_93M86	Collared	98.82	39.4446	Male
Sample_93M91	Collared	98.81	38.4341	Male
Sample_15F129	Collared	98.71	42.9708	Female
Sample_15F130	Collared	98.76	34.986	Female
Sample_15F131	Collared	98.75	42.3407	Female
Sample_15F135	Collared	98.74	39.3722	Female
Sample_15F142	Collared	98.65	37.9534	Female
Sample_15F143	Collared	98.69	36.4196	Female
Sample_15F145	Collared	98.73	41.3196	Female
Sample_15F149	Collared	98.71	39.559	Female
Sample_15F151	Collared	98.6	40.7103	Female
Sample_15F17	Collared	98.67	39.1225	Female
Sample_15F18	Collared	98.75	44.8449	Female
Sample_15F21	Collared	98.74	37.6521	Female
Sample_15F22	Collared	98.69	39.0517	Female
Sample_15F23	Collared	98.7	37.6489	Female
Sample_15F24	Collared	98.74	39.5439	Female
Sample_15F25	Collared	98.66	37.2903	Female
Sample_15F29	Collared	98.74	42.067	Female
Sample_15F447	Collared	98.74	38.4772	Female
Sample_15F448	Collared	98.77	48.7619	Female
Sample_15F450	Collared	98.74	50.5921	Female
Sample_15F453	Collared	98.69	32.2886	Female
Sample_15F457	Collared	98.74	34.3193	Female
Sample_15F459	Collared	98.76	38.7127	Female
Sample_15F460	Collared	98.76	41.3721	Female
Sample_15M153	Collared	98.71	44.2226	Male
Sample_15M155	Collared	98.72	30.8711	Male
Sample_15M158*	Collared	98.44	30.9622	Male
Sample_15M160	Collared	98.66	41.902	Male
Sample_15M161	Collared	98.63	43.5776	Male
Sample_15M162	Collared	98.67	44.81	Male
Sample_15M163	Collared	98.74	36.7124	Male
Sample_15M201	Collared	98.72	36.2441	Male
Sample_15M202	Collared	98.77	35.0701	Male

Sample_15M203	Collared	98.77	43.3666	Male
Sample_15M204	Collared	98.77	44.2558	Male
Sample_15M207	Collared	98.81	37.2538	Male
Sample_15M468	Collared	98.76	39.852	Male
Sample_15M469	Collared	98.88	43.8545	Male
Sample_15M475	Collared	98.76	32.956	Male
Sample_15M477	Collared	98.84	39.5779	Male
Sample_15M49	Collared	98.81	37.8717	Male
Sample_15M537	Collared	98.81	39.4165	Male
Sample_15M568	Collared	98.8	42.684	Male
Sample_15M571	Collared	98.92	38.6053	Male
Sample_15M573	Collared	98.85	46.054	Male
Sample_15M589	Collared	98.83	33.7914	Male
Sample_15M684	Collared	98.79	52.2221	Male
Sample_15M724	Collared	98.72	32.4109	Male
SP_11_M	Pied	85.8	33.1806	Male
SP_12_M	Pied	88.57	27.9139	Male
SP_13_M	Pied	87.24	34.7575	Male
SP_14_M*	Pied	91.29	43.4755	Male
SP_15_M	Pied	89.52	38.9494	Male
SP_16_F	Pied	94.81	24.3245	Female
SP_SV1_M	Pied	94.12	24.8969	Male
SP_SV2_M	Pied	91.78	29.2199	Male
SP_SV4_M	Pied	84.9	28.8335	Male
SP_SV5_M	Pied	88.48	30.0519	Male
SP_SV7_F	Pied	92.06	28.2795	Female
Sample_31	Red-breasted	79.9	44	Male
Sample_32	Red-breasted	95.51	21.88	Female
Sample_4	Red-breasted	94.83	52.66	Female
Sample_48	Red-breasted	51.29	42.37	Male
Sample_49	Red-breasted	94.16	51.41	Male
Sample_51	Red-breasted	94.47	44.74	Male
Sample_52	Red-breasted	94.69	47.59	Male
Sample_53	Red-breasted	94.82	34.63	Female
Sample_54	Red-breasted	95.18	26.17	Female
Sample_55	Red-breasted	95.46	48.4	Female
Sample_56	Red-breasted	96.22	53.12	Male
Sample_77	Red-breasted	95.31	35.82	Female
Sample_82	Red-breasted	95.49	42.17	Male
Sample_83*	Red-breasted	95	37.1	Male
Sample_92	Red-breasted	95.71	29.74	Female
Sample_1	Taiga	97.96	24.14	Male
Sample_10	Taiga	97.36	77.44	Female

Sample_11	Taiga	97.54	47.67	Female
Sample_12	Taiga	97.16	55.37	Female
Sample_13	Taiga	97.72	20.21	Female
Sample_16	Taiga	97.58	38.52	Female
Sample_17	Taiga	97.34	36.77	Male
Sample_18	Taiga	97.56	61.81	Male
Sample_2	Taiga	98.04	42.03	Male
Sample_20	Taiga	97.93	55.1	Male
Sample_21	Taiga	97.69	51.47	Female
Sample_22	Taiga	97.17	41.53	Female
Sample_23	Taiga	97.66	46.16	Male
Sample_24	Taiga	97.85	52.47	Male
Sample_25	Taiga	97.7	39.76	Female
Sample_26	Taiga	97.47	47.96	Female
Sample_28	Taiga	97.83	37.92	Male
Sample_29	Taiga	97.69	52.06	Female
Sample_3*	Taiga	97.75	29.64	Male
Sample_30	Taiga	97.86	43.14	Female
Sample_33	Taiga	98	47.77	Female
Sample_34	Taiga	97.99	38.64	Male
Sample_35	Taiga	97.72	28.94	Male
Sample_36	Taiga	97.9	44.91	Male
Sample_37	Taiga	98.04	48.84	Male
Sample_38	Taiga	98.22	56.54	Male
Sample_39	Taiga	98.02	51.45	Male
Sample_40	Taiga	98.11	33.1	Female
Sample_41	Taiga	97.71	46.5	Male
Sample_42	Taiga	97.33	49	Male
Sample_44	Taiga	96.55	43.99	Female
Sample_45	Taiga	98.02	49.05	Male
Sample_5	Taiga	97.79	29.3	Male
Sample_57	Taiga	98.19	43.43	Male
Sample_58	Taiga	97.86	32.7	Female
Sample_59	Taiga	98.1	58.48	Male
Sample_6	Taiga	97.23	34.89	Female
Sample_60	Taiga	97.94	62.05	Female
Sample_61	Taiga	98.17	67.99	Male
Sample_63	Taiga	97.81	21.89	Male
Sample_64	Taiga	98.07	49.95	Female
Sample_66	Taiga	98.14	37.8	Male
Sample_67	Taiga	97.99	25.62	Male
Sample_68	Taiga	98.13	34.11	Female
Sample_69	Taiga	98.17	20.25	Male

Sample_7	Taiga	97.95	37.48	Female
Sample_70	Taiga	97.96	41.51	Female
Sample_71	Taiga	98.16	104.16	Female
Sample_72	Taiga	98.08	34.7	Female
Sample_73	Taiga	98.06	49	Female
Sample_74	Taiga	97.92	39.99	Female
Sample_75	Taiga	98.1	30.02	Female
Sample_76	Taiga	98.11	27.17	Female
Sample_78	Taiga	57.39	30.21	Female
Sample_79	Taiga	97.88	46.98	Female
Sample_8	Taiga	97.88	23.16	Male
Sample_80	Taiga	98.31	44	Male
Sample_81	Taiga	97.95	57.27	Female
Sample_86	Taiga	98.13	34.66	Female
Sample_87	Taiga	97.94	44.37	Female
Sample_88	Taiga	98.05	48.94	Female
Sample_89	Taiga	97.65	25.64	Female
Sample_9	Taiga	97.88	44.88	Female
Sample_90	Taiga	97.96	55.95	Male
Sample_91	Taiga	98.18	54.95	Female
F_hyperythra_X	Snowy-browed	72.63	13.5387	Male

Table S2: Estimates of diversity and PSMC historical population size on autosomes and the Z-chromosome. Shown are estimates of nucleotide diversity for all four species, estimated using all SNPs on the autosomes and the Z-chromosome separately (see Supplemental Table S3 for GC-conservative estimates). Diversity estimates are also provided after masking sites in exons , conserved non-coding elements and their respective 1kb flanking regions, to account for the impact of linked selection (No LS). PSMC historical population size estimates for all four species are shown for the last 1Mya.

	**Collared**	**Pied**	**Red-breasted**	**Taiga**
π_A	0.00291 [0.00291;0.00292]	0.00235 [0.00235;0.00236]	0.00297 [0.00297;0.00297]	0.00305 [0.00305;0.00306]
π_Z	0.00205 [0.00205;0.00206]	0.00153 [0.00153;0.00154]	0.00244 [0.00244;0.00245]	0.00261 [0.00261;0.00262]
π_Z/π_A	0.705 [0.703;0.706]	0.651 [0.649;0.653]	0.822 [0.821;0.825]	0.855 [0.853;0.858]
π_A (No LS)	0.00317 [0.00317;0.00318]	0.00256 [0.00256;0.00256]	0.00321 [0.003120;0.00321]	0.00332 [0.00332;0.00333]
π_Z (No LS)	0.00219 [0.00218;0.00219]	0.00164 [0.00164;0.00165]	0.00260 [0.00260;0.00261]	0.00278 [0.00277;0.00278]
π_Z/π_A (No LS)	0.689 [0.687;0.692]	0.640 [0.638;0.642]	0.812 [0.810;0.814]	0.835 [0.832;0.834]
**$PSMC_A$** (until 1Mya)	182371 [177155, 186660]	137778 [133726, 142001]	123549 [120645, 127617]	96630 [94756, 103304]
**$PSMC_Z$** (until 1Mya)	132586 [127659, 138730]	92983 [89281, 97015]	91653 [86859, 95831]	79408 [69768, 80976]
**$PSMC_{Z/A}$** (until 1Mya)	0.727 [0.691, 0.768]	0.675 [0.636, 0.707]	0.742 [0.707, 0.779]	0.822 [0.706, 0.831]

Table S3: Estimates of diversity on autosomes and the Z-chromosome for GC-conservative polymorphisms. Shown are estimates of nucleotide diversity for all four species, estimated using only GC-conservative SNPs on the autosomes and the Z-chromosome separately. Diversity estimates are also provided after masking sites in exons or conserved non-coding elements, to account for the impact of linked selection.

	**Collared**	**Pied**	**Red-breasted**	**Taiga**
π_A	0.000465 [0.000464;0.000465]	0.000370 [0.000370;0.000371]	0.000518 [0.000517;0.000518]	0.000511 [0.000510;0.000511]
π_Z	0.000334 [0.000331;0.000336]	0.000247 [0.000246;0.000249]	0.000437 [0.000435;0.000440]	0.000444 [0.00441;0.000447]
π_Z/π_A	0.718 [0.713;0.729]	0.668 [0.663;0.672]	0.845 [0.840;0.849]	0.869 [0.864;0.874]
π_A (No LS)	0.000511 [0.000510;0.000512]	0.000407 [0.000406;0.000408]	0.000564 [0.000563;0.000565]	0.000562 [0.000561;0.000563]
π_Z (No LS)	0.000358 [0.000355;0.000361]	0.000265 [0.000263;0.000268]	0.000472 [0.000469;0.000475]	0.000475 [0.000472;0.000479]
π_Z/π_A (No LS)	0.700 [0.695;0.706]	0.652 [0.647;0.657]	0.837 [0.831;0.842]	0.845 [0.838;0.851]

Table S4: Estimates of selection on protein-coding sequences for autosomes and the Z-chromosome. Shown are estimates of π_N/π_S, d_N/d_S, and ω_a for genes on the autosomes and on the Z-chromosome. All estimates are based on all sites.

	**Collared**	**Pied**	**Taiga**	**Red-breasted**
**π_N/π_S A**	0.186 [0.178;0.194]	0.197 [0.188;0.207]	0.175 [0.167;0.183]	0.175 [0.166;0.182]
**π_N/π_S Z**	0.201 [0.161;0.237]	0.287 [0.218;0.333]	0.209 [0.165;0.270]	0.178 [0.138;0.213]
**d_N/d_S A**	0.180 [0.173;0.186]			
**d_N/d_S Z**	0.191 [0.172;0.209]			
**ω_a A**	0.0506 [0.0423;0.0568]	0.0370 [0.0237;0.0521]	0.0903 [0.0822;0.0961]	0.0287 [0.0200;0.0396]
**ω_a Z**	0.0752 [0.0369;0.101]	-0.0204 [-0.0591;0.0428]	0.0729 [0.0402; 0.103]	0.0823 [0.0357;0.112]

Table S5: Number of nonsynonymous fixed differences overlapping with selective sweeps. Shown for each species are the numbers of nonsynonymous fixed differences identified between the two species comparisons that also show a significant CLR estimate, separately for autosomes and the Z-chromosome. The percent of total nonsynonymous fixed differences showing a sweep signature is given in parentheses for both chromosome types.

	**Collared**	**Pied**	**Taiga**	**Red-breasted**
**Autosomes**	11 (8.7%)	41 (32%)	210 (6.9%)	394 (11%)
**Z-chromosome**	0 (0%)	2 (2.4%)	6 (1.9%)	3 (0.96%)

Table S6: Number of outlier PBS peaks on autosomes compared to the Z-chromosome. Shown are the number of 200-kb windows with and without a PBS peak for all four species, for both the autosomes and the Z-chromosome. Odds ratio and *P*-value indicate the significance level of the overrepresentation on the autosomes based on Fisher exact tests.

	**Autosome**	**Z-chromosome**	**Odds ratio**	***P*-value**
**Collared PBS peak**	187	11	1.0	1
**Collared no PBS peak**	4509	273		
**Pied PBS peak**	189	9	1.3	0.64
**Pied no PBS peak**	4507	275		
**Red-breasted PBS peak**	247	8	1.9	0.071
**Red-breasted no PBS peak**	4449	276		
**Taiga PBS peak**	253	10	1.6	0.22
**Taiga no PBS peak**	4443	274		

Table S7: Density of fixed differences in neutral regions and functionally constrained regions on the autosomes vs. the Z-chromosome. Shown are the proportion of fixed differences for varying functional constraint on autosomes and the Z-chromosome between both collared and pied flycatchers and red-breasted and taiga flycatchers. The fixed differences are separated based on whether they are potentially functionally constrained (UTRs, CNEs, and zero-fold degenerate sites) versus putatively neutrally evolving (intronic regions, intergenic regions, and four-fold degenerate sites). For both species comparisons the results of a Z-test are presented.

	**Autosome**	**Z-chromosome**	χ^2	***P*-value**
**Collared and pied constrained**	0.355	0.344	$2.94 \cdot 10^{-34}$	1
**Collared and pied neutral**	0.645	0.656		
**Red-breasted and taiga constrained**	0.308	0.309	$5.86 \cdot 10^{-34}$	1
**Red-breasted and taiga neutral**	0.692	0.691		

Table S8: The number of shared SNPs on the autosomes vs. the Z-chromosome for both species comparisons. In parentheses, the number of shared SNPs are given as a percentage of the total number of sites that are polymorphic in either species within each comparison.

Chromosome	Collared vs. pied	Red-breasted vs taiga
**Autosomes**	2011245 (16%)	577784 (2.0 %)
**Z-chromosome**	16341 (3.0%)	16715 (1.3%)

Table S9: The number of outlier f_d windows on the autosomes vs. the Z-chromosome. Shown are the number of windows on the autosomes and the Z-chromosome that have significantly reduced f_d values compared to the genome-wide background for two population comparisons. The population abbreviations are Atlas flycatcher (A), Öland pied flycatcher (OP), Spanish pied flycatcher (EP), Öland collared flycatcher (OC), and Italian collared flycatcher (IC).

Comparison	Window type	Autosomes	Z-chromosome	Odds ratio	*P*-value
**((A, OP), OC)**	f_d outlier	166	111	0.057	$< 10^{-16}$
**((A, OP), OC)**	No outlier	4351	165		
**((A, EP), IC)**	f_d outlier	159	126	0.044	$< 10^{-16}$
**((A, EP), IC)**	No outlier	4328	150		

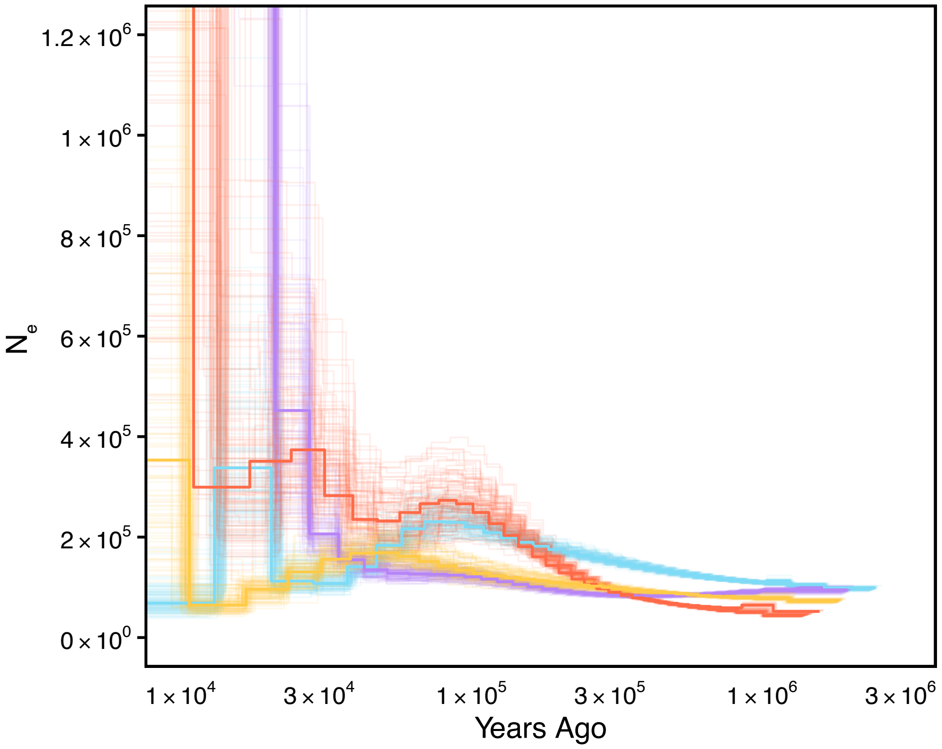

Figure S1: Historical changes in population size for all four species estimated on the Z-chromosome. One individual for each of the four flycatcher species is represented: collared flycatcher in blue, pied flycatcher in purple, red-breasted flycatcher in yellow, and taiga flycatcher in orange. The bold line represents the genome-wide estimate, bootstrap replicates are shown in lighter colour.

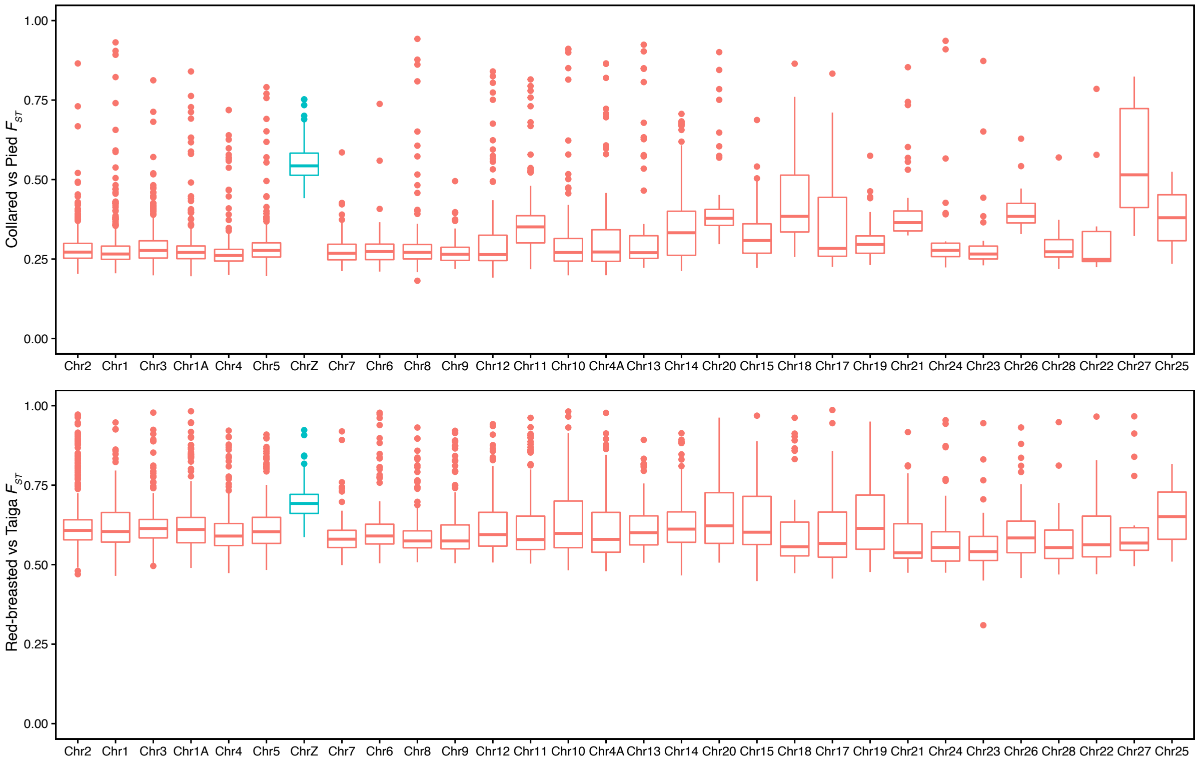

Figure S2: Distributions of F_{ST} estimates across chromosomes. Shown are estimates of F_{ST} in 200kb genomic windows for the collared and pied flycatchers and for the red-breasted and taiga flycatchers divided by chromosome. Chromosomes are ordered by decreasing size, with autosomes in red and the Z-chromosome highlighted in blue.

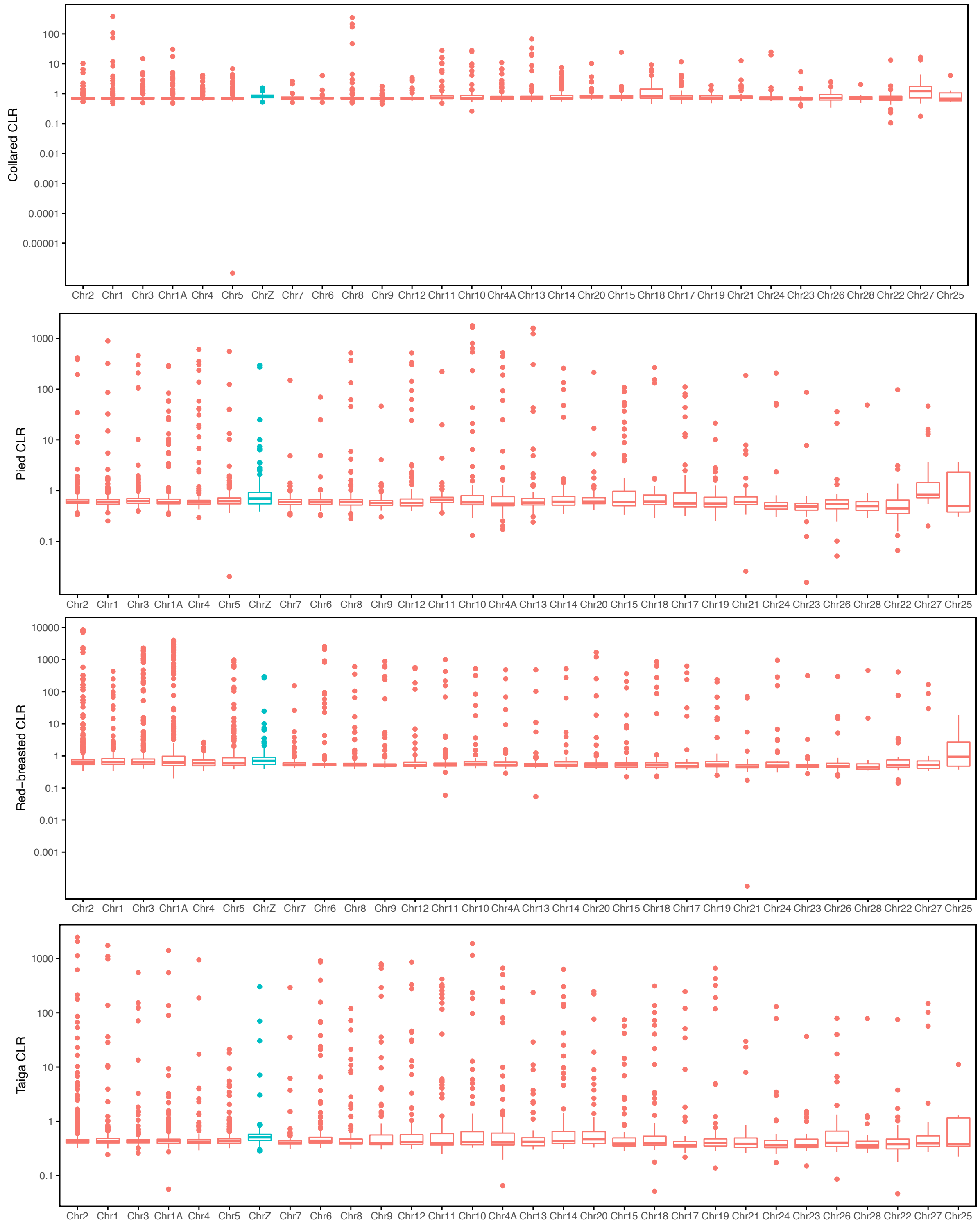

Figure S3: Distributions of CLR estimates across chromosomes. Shown are average CLR values in 200kb genomic windows for the four species, collared flycatcher, pied flycatcher, red-breasted flycatcher, and taiga flycatcher divided by chromosome. Chromosomes are ordered by decreasing size, with autosomes in red and the Z-chromosome highlighted in blue.

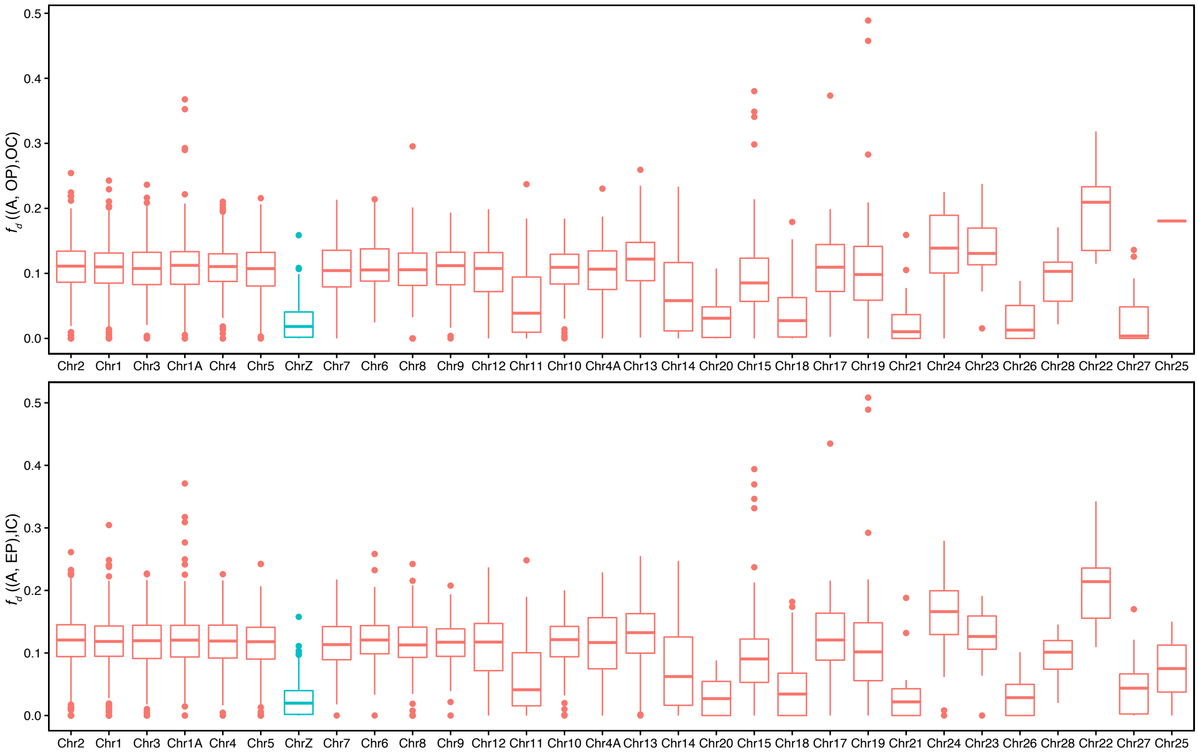

Figure S4: Distributions of f_d estimates across chromosomes. Shown are estimates of the f_d statistic in 200kb genomic windows for two population comparisons, divided by chromosome. The population abbreviations are: Atlas flycatcher (A), Öland pied flycatcher (OP), Öland collared flycatcher (OC), Spanish pied flycatcher (EP), and Italian collared flycatcher (IC). Chromosomes are ordered by decreasing size, with autosomes in red and the Z-chromosome highlighted in blue.
